# Open science interventions to improve reproducibility and replicability of research: a scoping review

**DOI:** 10.1098/rsos.242057

**Published:** 2025-04-09

**Authors:** Leonie Dudda, Eva Kormann, Magdalena Kozula, Nicholas J. DeVito, Thomas Klebel, Ayu P. M. Dewi, René Spijker, Inge Stegeman, Veerle Van den Eynden, Tony Ross-Hellauer, Mariska M. G. Leeflang

**Affiliations:** ^1^ Department of Otorhinolaryngology and Head & Neck Surgery, University Medical Center Utrecht, Utrecht, The Netherlands; ^2^ Brain Center, University Medical Center Utrecht, Utrecht, The Netherlands; ^3^ Open and Reproducible Research Group, Know Center GmbH, Graz, Austria; ^4^ Katholieke Universiteit Leuven, Leuven, Flanders, Belgium; ^5^ Nuffield Department of Primary Care Health Sciences, University of Oxford, Oxford, UK; ^6^ Epidemiology and Data Science, Amsterdam UMC Locatie AMC, Amsterdam, Noord-Holland, The Netherlands; ^7^ Cochrane Netherlands, University Medical Center Utrecht, Utrecht, The Netherlands; ^8^ Medical Library, Amsterdam UMC Locatie AMC, Amsterdam, Noord-Holland, The Netherlands; ^9^ Graz University of Technology, Graz, Austria

**Keywords:** open science, reproducibility, scoping review

## Abstract

Various open science practices have been proposed to improve the reproducibility and replicability of scientific research, but not for all practices, there may be evidence they are indeed effective. Therefore, we conducted a scoping review of the literature on interventions to improve reproducibility. We systematically searched Medline, Embase, Web of Science, PsycINFO, Scopus and Eric, on 18 August 2023. Any study empirically evaluating the effectiveness of interventions aimed at improving the reproducibility or replicability of scientific methods and findings was included. We summarized the retrieved evidence narratively and in evidence gap maps. Of the 105 distinct studies we included, 15 directly measured the effect of an intervention on reproducibility or replicability, while the remainder addressed a proxy outcome that might be expected to increase reproducibility or replicability, such as data sharing, methods transparency or pre-registration. Thirty studies were non-comparative and 27 were comparative but cross-sectional observational designs, precluding any causal inference. Despite studies investigating a range of interventions and addressing various outcomes, our findings indicate that in general the evidence base for which various interventions to improve reproducibility of research remains remarkably limited in many respects.

## 1. Introduction

The reliability and trustworthiness of research results are in question [1–3]. This is true especially with respect to their *reproducibility* (defined in this paper as obtaining the same or similar results when rerunning analyses from previous studies using the original design, data and code; cf. [4]) and *replicability* (defined here as obtaining the same or similar results when repeating, in whole or part, a prior study; cf. [4]). Ensuring that the results of studies can be independently confirmed, is expected to reduce waste [2,5] and lead to more reliable outcomes that better inform evidence-based decisions [6,7]. Furthermore, studies that can be independently confirmed may increase public trust in the scientific enterprise [8,9]. Reproducibility and replicability therefore underpin the credibility and reliability of research findings in many fields, especially in science, technology, engineering and mathematics. For the sake of brevity, we use the term ‘reproducibility’ to include ‘reproducibility’ and ‘replication’, as well as the reproducibility of methods or code, etc. (i.e. what Goodman *et al*. [10] call ‘methods reproducibility’). Where more narrow usage of terms, per Nosek *et al*. [4], is intended, we will state this explicitly.

Debates on reproducibility have gained increasing prominence in various scholarly fields, as well as in the general press [11,12]. Pivotal to this debate were failures to reproduce study findings across the medical, behavioural and social sciences. Fields like psychology [13], biomedical research [14,15], economics [16] and broader social sciences [17] all witnessed ‘many-lab’ studies whose findings indicated levels of reproducibility ranging between 30 and 70%. A piece in *Nature News* in 2016 reported survey findings (ironically themselves lacking in transparency) which highlighted that between 60 and 80% of scientists across various disciplines encountered hurdles in reproducing the work of their peers, with similarly noteworthy difficulties encountered when attempting to replicate their own experiments (40–60%) [1]. Other scholars have argued that the meanings and relevance of ‘reproducibility’ vary widely across research fields and point to the unsuitability of these concepts—at least as strictly as it is understood in more experimental domains—as a criterion of quality within the humanities [18–20] or qualitative research [21–24].

Given these interdisciplinary differences, factors and practices that may influence the level of reproducibility of research must be expected to vary in their effectiveness (what works, under what circumstances). Factors that have been associated with perceived poor levels of reproducibility include selective non-publication, questionable research practices, insufficient training in research methods and a lack of transparency and data accessibility [5,25–27]. Interventions to improve reproducibility may target these practices. Proponents of open science believe that openness of methods, materials and community will improve reproducibility of science [28]; however, inadequate access to the necessary data for rerunning experiments or analyses remains a major concern throughout science [1,29–31].

Although multifaceted efforts underscore a shared commitment towards reproducibility within much of the academic landscape, the extent to which practices and interventions have been empirically investigated for their effectiveness to improve reproducibility, across research disciplines and time, remains unclear [32]. To assess which interventions have been formally tested for their effectiveness in improving the reproducibility of science, we conducted a scoping review of the literature. By summarizing current evidence and highlighting gaps in knowledge, this study aims to provide insights for researchers, policymakers, and stakeholders invested in promoting reproducibility.

### 1.1. Objectives

The objective of this scoping review is to provide an overview of interventions that have been formally investigated for their effect on reproducibility and replicability in science. An intervention could be any action exhibited by either individual researchers or scientific institutions (e.g. research institutes, publishers and funders) with the aim to enhance reproducibility. We specifically focused on open science practices but interpreted their definition broadly.

## 2. Methods

### 2.1. Study registration

The study protocol was uploaded to the Open Science Framework (OSF) (doi: 10.17605/OSF.IO/D65YS) prior to data collection and published on Open Research Europe [33]. Deviations from the protocol are reported transparently in the electronic supplementary material, S1. All materials, including the data-extraction sheets, data and code are available on OSF (doi: 10.17605/OSF.IO/7EF5H).

### 2.2. Design: scoping review

Scoping reviews, also sometimes referred to as ‘mapping reviews’ or ‘scoping studies’, are particularly helpful when performing evidence synthesis on complex and heterogeneous areas [34]. They usually address broader review questions than traditionally more specific systematic reviews. As the notion of reproducibility and replicability varies across disciplines, we gathered a multidisciplinary team of authors and based the terms used throughout the scoping review on broad definitions informed by multiple sources across disciplines. We conducted the scoping review following the guidance provided by the Joanna Briggs Institute, and we reported the scoping review following the Preferred Reporting Items for Systematic Reviews and Meta-Analyses: extension for Scoping Reviews (PRISMA-ScR) guidelines (see the electronic supplementary material, S6) [35].

### 2.3. Eligibility criteria

#### 2.3.1. Study designs

We included studies evaluating the effectiveness of interventions aimed at enhancing the reproducibility or replicability of scientific methods and findings. These should have a comparative design, either between-subject or within-subject comparisons. Non-comparative studies were only included if the introduction of an intervention was explicitly stated (e.g. post-intervention study reporting the level of reproducibility, or related practices, after the implementation of a guideline). Studies that only report the prevalence of a practice or behaviour (e.g. measuring only the amount of pre-registration in a field) without any clear connection to a discrete action that could have impacted the rates, nor a clear link to a reproducibility-related outcome, were excluded.

We excluded studies if they did not test an intervention, if the full text was unavailable and if the article did not report primary findings, such as stage 1 registered reports or position papers. We also excluded studies that investigated agreement in test results or diagnoses between observers or laboratories, which is also sometimes referred to as ‘reproducibility’.

#### 2.3.2. Article types

We included any article type containing primary data, including peer-reviewed original articles, early access papers and preprints. Position papers, study protocols, editorials and any other articles lacking primary data were excluded. Reviews were also excluded from our final synthesis, but their reference lists were checked for additional relevant publications. We used Zotero reference management software to check for any retracted studies within our corpus of articles (none were found).

#### 2.3.3. Participants

Participants in eligible studies could be researchers of any career level, holders of other academic posts such as editors or administrative staff, or an entire academic institution such as a university, journal, publisher or funder. The included studies could cover any scientific field or discipline.

#### 2.3.4. Interventions and comparators

We included any intervention that aimed to improve the reproducibility and replicability of science, basing the categories of interventions on broad definitions taken from multiple disciplines. A list of interventions that we expected to include are listed in the protocol [33] and in the electronic supplementary material, S4. We included studies with any comparator.

#### 2.3.5. Outcomes

Our primary outcome is the reproducibility or replicability of research. This means studies that attempted to replicate or reproduce another study, including aspects such as data collection, analyses and/or conclusions (electronic supplementary material, S4).

Since studies directly examining replicability or reproducibility were expected to be rare, we also defined a number of proxy outcomes that would be necessary, but not necessarily sufficient, for improved reproducibility. These proxies—practices commonly claimed within the literature to support reproducibility—were defined using a conceptual framework drawn from key texts [25,29,30,36], along with expert consultation on the measurement and assessment of reproducibility from different disciplines. Based on these considerations, outcomes such as pre-registration, data sharing and completeness of reporting were therefore considered to be within the scope of the review as proxies that could facilitate reproducibility. As a consequence, various open science practices were included both as interventions and as proxy outcomes for reproducibility.

### 2.4. Search strategies

#### 2.4.1. Development of the search query

The development of the search strategy was an iterative process, containing multiple steps and feedback loops. As the notion of reproducibility and replicability varies across disciplines, we based the search terms for interventions and outcomes on broad definitions taken from multiple texts. We searched 10 databases to ensure coverage across a variety of disciplines.

First, we requested ‘key articles’ based on the inclusion criteria from the study team, and a broader group of experts, to develop and validate the search queries. This process initially identified five key articles. We then used the Connected Papers tool (https://www.connectedpapers.com/) to identify an additional eight key articles. Throughout this phase, the number of articles was progressively reduced as we refined and discussed the inclusion criteria. Ultimately, only interventional articles were included to establish a definitive set of key articles. The intent was to base the search on articles that were unequivocally within scope, rather than those that were only partially relevant. This approach underscores the complexity of the project and highlights the challenge of defining clear boundaries within research definitions. The 15 key articles are available in a public Zotero folder (https://www.zotero.org/groups/4983756/osirisconsortium/collections/5Q3IJKZ2) and in a .ris file on the project OSF page (https://osf.io/7ef5h/).

#### 2.4.2. Electronic search

The electronic searches were performed on 18 August 2023. We systematically searched Medline, Embase, Web of Science, PsycINFO, Scopus and Eric. All databases were searched from inception until 18 August 2023. The search string was initially developed for Medline and then translated to the other databases. An initial search was constructed to align with our research group’s understanding of the research question, yielding 16 930 references for screening. Following this initial screening, it was evident that certain topics were inadequately covered. Minor adjustments to the search strategy were implemented, resulting in an additional 472 references. After screening this initial batch, we discussed the outcomes and determined that the search still did not adequately capture all areas of this complex and cross-disciplinary research area. Consequently, a more sensitive search was developed to encompass the under-represented topics from the initial results, aiming to achieve more comprehensive coverage. Terms that were added at this stage referred to reporting checklists and more qualitative research. This final search, while largely overlapping with the initial searches, introduced 18 661 additional unique references leading to a total of 36 063 items for title and abstract screening. The final search strings are available in the electronic supplementary material, S2.

#### 2.4.3. Other searches

We undertook an independent search for grey literature by contacting colleagues in October and November 2023, with e-mail requests for any relevant non-academic publications that address reproducibility interventions.

### 2.5. Selection process

All search results were collected in EndNote 20.6, and duplicate references were removed using the software’s deduplication tool (http://dedupendnote.nl/, v. 1.0.0). Any remaining duplicated references were excluded manually during screening in EPPI Reviewer 6. At least two authors independently screened the reference lists of relevant reviews identified during screening for additional studies for inclusion. Records identified through any aspect of our search strategy were first screened for eligibility based on the title and abstract and then by reading the full manuscript. The exact process is described in further detail below.

#### 2.5.1. Title and abstract screening

Title and abstract screening were performed by L.D., E.K., M.K., N.J.D., T.R.-H., V.V.d.E. and M.M.G.L. using EPPI Reviewer software ([37], v. 6). Initially, we planned to do the title and abstract screening in duplicate. However, owing to the large volume of articles, we opted for a single screening approach. Each assessor marked each title and abstract as *Include*, *Exclude* or *Maybe*. The articles marked as *Maybe* were then double-checked by a senior author.

#### 2.5.2. Full-text screening

For articles that passed title and abstract screening, the full text was retrieved and reviewed by at least two members of the study team independently to determine final eligibility for the review. Full-text screening was conducted by authors L.D., E.K., M.K., N.J.D, T.K., A.P.M.D, V.V.d.E., T.R.-H., M.M.G.L. and additional contributors (see Acknowledgements). Disagreements during screening were rectified by the senior authors T.R.-H., M.M.G.L. and V.V.d.E., with additional discussion with the broader study team as needed.

To reduce the differences in screening between the authors, we started with a pilot screening of 23 randomly selected articles that were reviewed by the entire study team and then discussed. As new reviewers were added to the project, they completed a modified version of this pilot screening focusing on a selection of 10 articles from the original 23. If a manuscript was in a foreign language, we either assigned the article to a team member who spoke that language or used translation tools (Google Translate (https://translate.google.com) or DeepL translator (https://www.deepl.com/en/translator)) on the full text to assess eligibility.

### 2.6. Data extraction

A draft data-extraction sheet, including extraction instructions and terminology definitions, was developed by L.D., M.K. and T.R.-H. and reviewed by the whole team. Data extraction was done by L.D., E.K., M.K., N.J.D, T.K., A.P.M.D, V.V.d.E, T.R.-H., M.M.G.L and additional contributors (see Acknowledgements). All extracting authors tested the extraction sheet using 10 articles and revised the final sheets based on pilot feedback. Fields extracted from included articles are detailed in the electronic supplementary material, S3. Data extraction was not conducted independently in duplicate as outlined in our protocol, primarily owing to time constraints, but all extractions were double-checked by another author to ensure consistency and validity. Data extraction took place in the EPPI Reviewer. Owing to time constraints, we did not contact authors in case of insufficient data. If an article addressed multiple research questions referring to different outcomes, we separately extracted the data for each question. We therefore use the term ‘article’ for the actual publication, and ‘study’ for the separate outcomes that may be contained within a given ‘article’.

### 2.7. Data synthesis and analysis

The data collected through the EPPI Reviewer were transferred to and cleaned in Microsoft Excel. We also used Microsoft Excel to summarize the characteristics of the included studies according to the interventions applied, the outcomes measured, the study design, and the stakeholders involved. The data extracted from the articles were presented graphically in evidence gap maps, which are a form of bubble plot with the different outcomes on the *x*-axis and the interventions on the *y*-axis. The size of the bubbles was defined by the number of studies that address this particular intervention/outcome combination and the colour of the bubbles was defined by the study design. If studies with different designs addressed similar intervention-outcome combinations, this was depicted using multiple colours. Information about stakeholders and academic field was represented using bar charts. We added extra variables to our dataset to enable more detailed analyses (e.g. exact wording of interventions and outcomes as used by the authors) than were possible with the initial data-extraction items and we renamed variables to facilitate labelling (e.g. intervention classification was changed into intervention_class; study design category was changed into study_design) in graphs and tables (electronic supplementary material, S3–S5). All graphs were made using ggplot2 v. 3.5.0 in R [38,39].

## 3. Results

Initially, we retrieved 36 063 articles; 1643 of these passed the title and abstract screening. Following the full-text screening, 172 articles were included for data extraction. From the reference lists of relevant reviews, we assessed the full texts of an additional 42 articles, of which we included three for data extraction. During data extraction, 88 further articles were excluded, because they were deemed out of scope. Most of these did not test a formal intervention (*n* = 27) or assess an irrelevant outcome (*n* = 24), such as the power of a study or whether a guideline could be retrieved.

**Figure 1.**
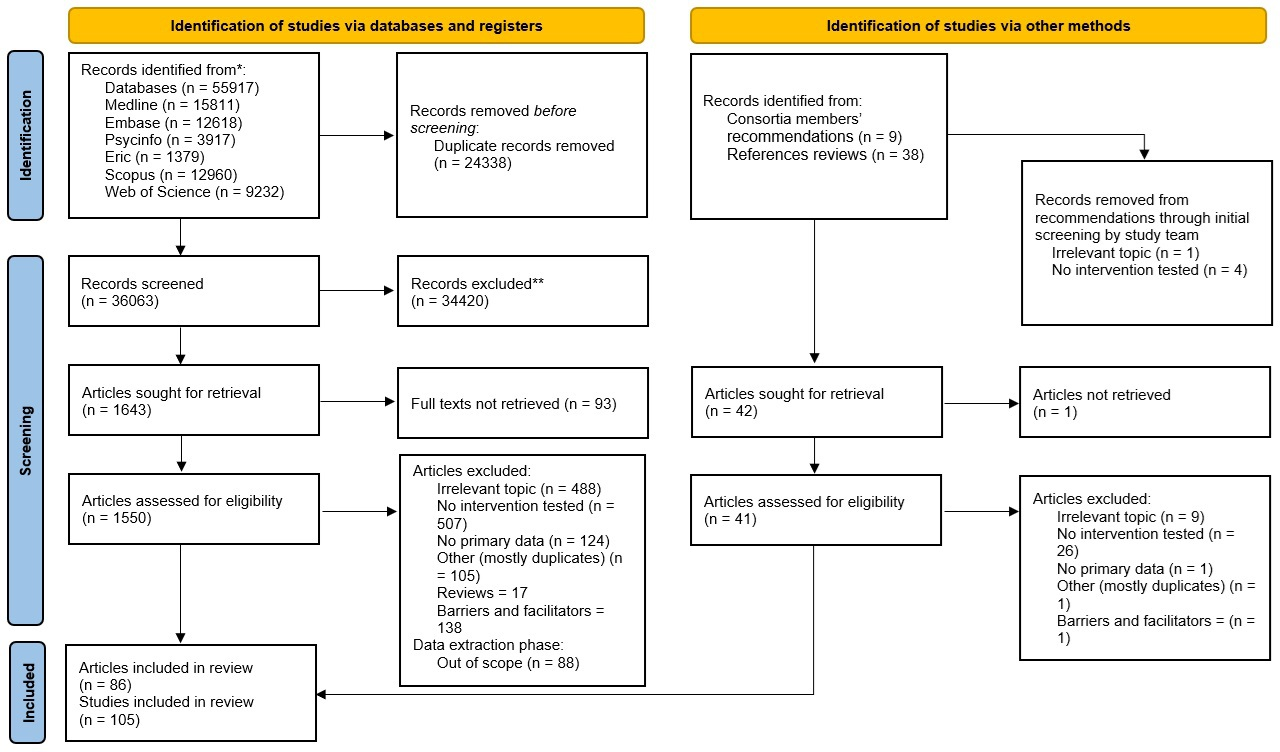
PRISMA flowchart of the search and selection process. The boxes show the numbers of retrieved, excluded and sifted articles in each step of the searching, screening and selection process. In the end, we included 86 articles, containing 105 studies.

We thus included data from 86 articles in this scoping review (figure 1; [31,40–124]). Most of these (*n* = 69) assessed one intervention and one outcome; however, 17 presented data for two (*n* = 15) or three (*n* = 2) separate hypotheses, separate research questions, separate sub-studies or separate specific interventions or outcomes. Therefore, our analyses were based on 105 studies from 86 articles. Of these 105 studies, only 15 directly assessed the effect of one or more interventions on reproducibility or replicability by re-analysing data or actually reproducing the results of previous research. The remaining 90 studies investigated the effect of one or more interventions on a proxy outcome, such as data sharing or complete reporting. A glossary of definitions can be found in the electronic supplementary material, S6.

### 3.1. Characteristics of the included studies

Table 1 shows the characteristics of the included studies. Of the 86 included articles, 85 (99%) were peer reviewed; we included one non-peer-reviewed preprint. One peer-reviewed editorial about the implementation of registered reports by the editorial board was included as it presented original data. Although we included all languages, the language of publication of the overwhelming majority of our included articles is English, with one editorial written in German. The included studies used a variety of study designs, as described below.

**Table 1.** Characteristics of included studies (*n* = 105).

characteristic	category	*n*	%
study design	randomized experiment	5	4.77
	comparative (between-subject, non-randomized)	61	58.09
	comparative (within-subject)	7	6.66
	post-intervention only	25	23.81
	demonstration of a tool	6	5.71
	other—individual patient meta-analysis	1	0.95
academic discipline	medical and health sciences	58	55.24
	social sciences	22	20.95
	natural sciences	5	4.76
	medical and health sciences and social sciences	6	5.71
	medical and health sciences and natural sciences	8	7.62
	social sciences and natural sciences	1	0.95
	multidisciplinary	5	4.76
intervention type	open methodology	10	9.52
	rewards and incentives	9	8.57
	reporting guidelines, reporting standards	15	14.29
	policy guidelines (e.g. of funders and journals)	49	46.67
	open data and materials	2	1.90
	training and education	3	2.86
	open science tools	7	6.67
	other	10	9.52
intervention implementer	journal or publisher	63	60.00
	academic staff	17	16.19
	institution	4	3.81
	peer reviewer	2	1.90
	government	1	0.95
	multiple or other entities	6	5.71
	not applicable	12	11.43
outcome type	reproducibility	9	8.57
	replicability	2	1.90
	other direct outcomes	4	3.81
	registration status	4	3.81
	methods transparency	36	34.29
	research material sharing	34	32.38
	other	16	15.24
author’s conclusion	generally positive	60	57.14
	null/neutral	44	41.90
	generally negative	1	0.95

Randomized controlled trial (*n* = 6 studies): five studies randomized subjects to receive the intervention or not [48,55,78,102,115]. One study randomized six sections within a manuscript, with authors randomly received instructions for three out of six sections, or for none of the sections in the control group [46].

Comparative between-subject design, cross-sectional (*n* = 27 studies): these studies compared at one point in time (or during a fixed time frame) participants exposed to a certain intervention with participants who had not been exposed to this intervention [51,66,72,76,83,87–89,92,93,95,100,106,111,112,114,118–121,123,124]. In four studies [51,76,95,114], the interventions were experimentally applied by the research teams themselves. In five other studies, the intervention was not directly applied by the study team, as it consisted of a generic policy brief or a publication by a third party. In all other cross-sectional studies, a journal, publisher or funder applied the intervention.

Comparative between-subject design, longitudinal (*n* = 11 studies): these 11 studies measured the outcome at multiple different time points in different subjects (often articles), for example the measurement of reporting in articles over multiple years after the introduction of a journal reporting [43,64,65,77,99,101,103,117]. One study followed a group of researchers who built a collaborative training community [101]. All 11 studies were observational in nature.

Comparative between-subject design, pre-post intervention (*n* = 21 studies): this design was often applied in studies that checked the reporting status of studies or materials- and methods-sharing status of studies before and after a certain event, for instance, a change in journal policy [40,41,47,49,50,54,61,63,75,79,81,84,85,91,96,104,122].

Comparative between-subject design, non-randomized experimental (*n* = 1): one study [116] experimented with a tool to help authors to adhere writing guidelines. The authors could choose whether they would use the tool or not.

Comparative within-subject design (*n* = 7 studies)*:* these seven studies used a variety of designs, from measuring behavioural changes after a workshop [59] and standardization within one institute [109], to re-analyses of statistical findings ([69,70] with two studies, [119]). One study [62] did not report any quantitative results, instead, the authors described the experiences after the implementation of an intervention. In all seven, the intervention was intentionally applied in an experimental setting.

Observational post-intervention-only design (*n* = 25 studies): this design was often applied in studies that checked the reporting status of studies or materials- and methods-sharing status of studies after, e.g. the impact of journal policy on these areas. There was no baseline measurement reported before the implementation of the intervention [31,44,45,47,53,56–58,60,67,68,72–74,90,94,97,98,105,107,108,110].

Demonstration of a tool (*n* = 6 studies): these are studies that apply a certain tool, technique, statistical solution or workflow to one or more use cases [42,52,80,82,86,113]. These studies are often limited to one project team, applying the intervention to themselves.

Other: there was one individual participant meta-analysis [71], which investigated associations between data availability statements and actual data availability, and associations with several other factors (e.g. journal policy, type of data, trial design and human participants).

The unit of analysis for 77 of the 105 included studies was a published article (of which 13 were limited only to clinical trials). In these 77 studies, the number of articles investigated ranged from 3 to 2 121 580. Other units of analysis included were abstracts, analyses, datasets, institutions and individual researchers.

### 3.2. Interventions

The grouping of interventions was difficult owing to nuanced differences in apparently similar topics. For example, some studies examining reporting guidelines investigated whether these guidelines were mentioned in the authors’ guidelines, while others examined whether journals actively requested completed checklists. Similarly, data-sharing studies could focus on data-sharing statements or actual data availability in a repository.

Some interventions could be seen as an exposure instead. For example, a board of editors issuing a statement about data sharing does not directly intervene in the research process but can still influence behaviour. This type of ‘exposure’ could have—in principle—been subjected to randomized evaluations (e.g. journals randomly adopting a policy or not). Other interventions in our list, such as retractions or the introduction of published guidelines cannot be randomized. For the purpose of this scoping review, we considered all these factors ‘interventions’. We grouped the interventions as explained in table 2.

**Table 2.** List of interventions implemented in the included studies (*n* = 105).

intervention class and subclasses	*n*	%	intervention specifics	*n*	%
open methodology					
open protocol	1	0.95	publication of protocol	1	0.95
pre-registration, study registration, analysis plan	5	4.76	pre-registration	5	4.76
registered reports	2	1.90	registered reports	2	1.90
project workflow	2	1.90	standardization of processes	2	1.90
open science policies and guidelines					
rewards and incentives	9	8.57	badges for data and code sharing	2	1.90
			badges for open data	3	2.86
			badges for open data and materials	1	0.95
			badges for open science	3	2.86
reporting guidelines, reporting standards	15	14.29	use of reporting checklist	3	2.86
			publication of reporting checklist	11	10.48
			publication of methodological guidelines	1	0.95
policy guidelines (e.g. of funders/ publishers)	49	46.67			
— on data/code sharing or open science practices			journal submission guidelines on data/code sharing	3	2.86
			journal policy on data/code sharing	20	19.05
			ICMJE data-sharing statement	5	4.76
			journal and funder policies on data/code sharing	1	0.95
			funder data-sharing policy	1	0.95
			journal policy strictness (data/code sharing)	1	0.95
— on research quality			journal submission guidelines on methodology	2	1.90
— on acceptance and registration before study has started			journal accepts regardless outcome	1	0.95
			journal policy on trial registration	3	2.86
			ICMJE proposed trial registration	2	1.90
			journal submission guidelines on pre-registration	1	0.95
			FDA trial registration policy	1	0.95
— on reporting checklists or guidance			journal endorses reporting checklist	4	3.81
			ICMJE endorses reporting guideline	1	0.95
			journal requires reporting checklist	2	1.90
			journal submission guidelines on reporting guidelines	1	0.95
open data and materials					
data availability	1	0.95	authors shared data	1	0.95
affirmative sharing declarations	1	0.95	data-sharing statements	1	0.95
open educational resources					
training	2	1.90	training	2	1.90
other	1	0.95	results-free review	1	0.95
open science tools					
open workflow tools, workflow management systems	1	0.95	tools	1	0.95
dockerization, docker	1	0.95	statistical tool	1	0.95
other	5	4.76	automatic code cleaning	1	0.95
			standardization of processes	1	0.95
			writing tool	3	2.86
other interventions					
type1/type 2 error reduction	3	2.86	effect size filter	1	0.95
			*p*-value calibration	2	1.90
collaborative research	1	0.95	building a (training) community	1	0.95
other	6	5.71	editorial checks/peer review	3	2.86
			role models	1	0.95
			discrepancy review	1	0.95
			statistical approach	1	0.95

Open methodology (*n* = 10): eight studies investigating some sort of pre-registration of research plans. Five studies investigated the effect of pre-registration [108,118] (three studies), [120], one the effect of publishing the protocol of a study [66] and two studies investigated registered reports [97,114]. Two other studies investigated the effect of open workflows [42,109].

Policy guidelines issued by publishers and journals (*n* = 46): the intervention most commonly studied was the implementation of a policy by publishers and journals. Seven of those investigated the effect of statements issued by the International Committee of Medical Journal Editors (ICMJE) on data sharing [47,58,105,110], trial registration [73,112] and reporting guidelines [44]. Twenty-five investigated journal policies or author instructions about data sharing [31,43,45,57,60,68,71,72,75,84,87,92,94–96,99,100,103,107,117,119]; seven about reporting guidelines [41,51,67,77,83,123,124] and four about pre-registration of studies, including trial registration [49,73,89,111]. Two studies from the same author investigated the effect of generic methodological journal guidelines with 5 years between the two studies [64,65].

Policy guidelines by funders or government agencies (*n* = 3): two studies investigated policy issued by funders, more precisely the requirement of the National Institutes of Health to provide a data-sharing plan for large funding grants [93,99]. One study investigated the effect of the United States Government’s trial registration policy [88].

Reporting guidelines (*n* = 15): fifteen studies evaluated the effect of reporting guidelines in general [40,50,51,53,54,61,63,79,85,91,95,98,106,121,122]. The baseline for these studies was the publication of a certain reporting checklist or guideline, and the design was either a pre-post design or a post-intervention-only design.

Rewards and incentives (*n* = 9): nine studies investigated the effect of badges on open science or data sharing [56,65,65,74,74,81,102,104]. Two of those investigated the effect of receiving a badge by comparing the amount of data shared from authors who have and have not received a badge, while the other seven compared outcomes between journals issuing badges or not.

Specific tools (*n* = 7): seven studies looked at specific tools, such as web-based writing or reporting aids (*n* = 3) [46,78,116], workflows and standardization tools (*n* = 2) [86,113] and statistical and automated code cleaning tools (*n* = 2) [52,119].

Other interventions (*n* = 15): other interventions included the building of an open science training community (*n* = 1) [101], specific training modules (*n* = 2) [59,90], results-free peer feedback (*n* = 1) [62], *p*-value adjustments or other statistical solutions (*n* = 4) [69,70,80], discrepancy reviews (*n* = 1) [67], data and materials sharing (*n* = 2) [47,82], interventions aimed at peer-reviewers or editors (*n* = 3) [48,55,115] and role models (*n* = 1) [55].

In 61 studies, the party issuing the intervention was the journal, and in 16 studies, academic staff applied the intervention. Academic staff were the intervention target in 95 studies, in most cases, they were only holistically referred to as ‘authors’. Two studies were aimed at peer reviewers, one at journals and one focused specifically on PhD students.

### 3.3. Interventions not retrieved

Of the 69 interventions included in our data-extraction sheet, 52 were not investigated in any of the included studies (electronic supplementary material, S4). These represent gaps in the evidence. Examples are interventions such as retractions and funding statements. Also, typical open science interventions, such as FAIR data, open science ethics, open peer review and open evaluations are lacking in studies investigating their effect on reproducibility.

### 3.4. Outcomes—direct reproducibility

Fifteen studies investigated the effect of an intervention on reproducibility or replicability as direct outcomes (table 3). One study investigated the effect of data-sharing policy on inferential reproducibility [47]. Eight studies investigated whether interventions improved the reproducibility of scientific results: one study investigated the effect of a statistical tool on whether analytic code could be reproduced [52]; two studies investigated the effect of badges on whether the results of a study could be reproduced without discrepancies [56,74]; two studies investigated the effect of journal policies on whether the results of a study could be reproduced without discrepancies [84,94]; one study investigated the effect of registered reports on whether the same main results with minimal mistakes could be reproduced [97]; one study investigated the effect of workflow tools on whether the same results could be reproduced [86]; and one study investigated the effect of building a training community on whether articles from this community could be reproduced [101]. Two studies investigated the effect of standardization of processes and statistical analyses on replicability of results from different laboratories [42,109]. One article included three studies about whether statistical inconsistencies could be solved by sharing the data [96], and one study investigated the effect of statistical intervention on the reduction of type I errors [80].

**Table 3.** List of outcomes investigated in the included studies (*n* = 105).

outcome domain	outcomes	*n*	%
reproducibility	inferential reproducibility	1	0.95
	results reproducibility	8	7.62
replicability	results replicability	2	1.90
other direct outcome	statistical inconsistencies	3	2.86
	type I error reduction	1	0.95
registration status	pre-registration	4	3.81
methods transparency	analysis transparency	1	0.95
	design transparency	1	0.95
	research process or workflow transparency	1	0.95
	methods transparency general	33	31.43
research material sharing	material sharing	1	0.95
	data sharing	29	27.62
	code sharing	3	2.86
	research material sharing general	1	0.95
other proxy outcome	other	16	15.24

### 3.5. Proxy outcomes

Eighty-nine studies assessed the effect of an intervention on a proxy outcome for reproducibility. Of these 89 studies, 36 assessed the effect of one or more interventions on the transparency of research or methods [40,41,44,46,48,50,51,53–55,61,63–65,67,77–79,83,85,90,91,95,98,106,111,115,116,118,121–123]. This outcome was most commonly assessed via a reporting checklist, such as CONSORT or ARRIVE, while the intervention was a policy about, or publication of, reporting standards. One study asked researchers to rate the perceived transparency of their research.

Another 26 studies assessed the effect of one or more interventions on data-sharing. Of these, 10 studies checked whether the data were actually freely available in a repository without first asking for it; four studies only checked whether a statement was made about data availability [60,75,93,110]; and 12 studies checked whether data were actually shared after a request [58,72,76,92,94,95,103,105,107,117]. The intervention in these studies was a policy on data or code sharing implemented by a journal or funder.

Other proxy outcomes were as follows: pre-registration of clinical trials and trial data sharing [89,112]; pre-registration of systematic reviews [120]; pre-registration of other study types [73]; how transparent researchers rated themselves after a training module [59]; and data or code sharing [45,57,72,84,104] after journal policy changes. Some of these outcomes, such as pre-registration, have also been included in other studies as interventions.

### 3.6. Outcomes not retrieved

Contrary to our initial list of interventions, most of the items on our initial list of outcomes were investigated. We did not retrieve any studies that directly investigated the effect of interventions on methodological or inferential reproducibility, and we did not retrieve any studies on the proxy outcomes registered reports, post-study peer review, reproducibility checks, data management plans and software sharing.

### 3.7. Evidence gap maps

**Figure 2.**
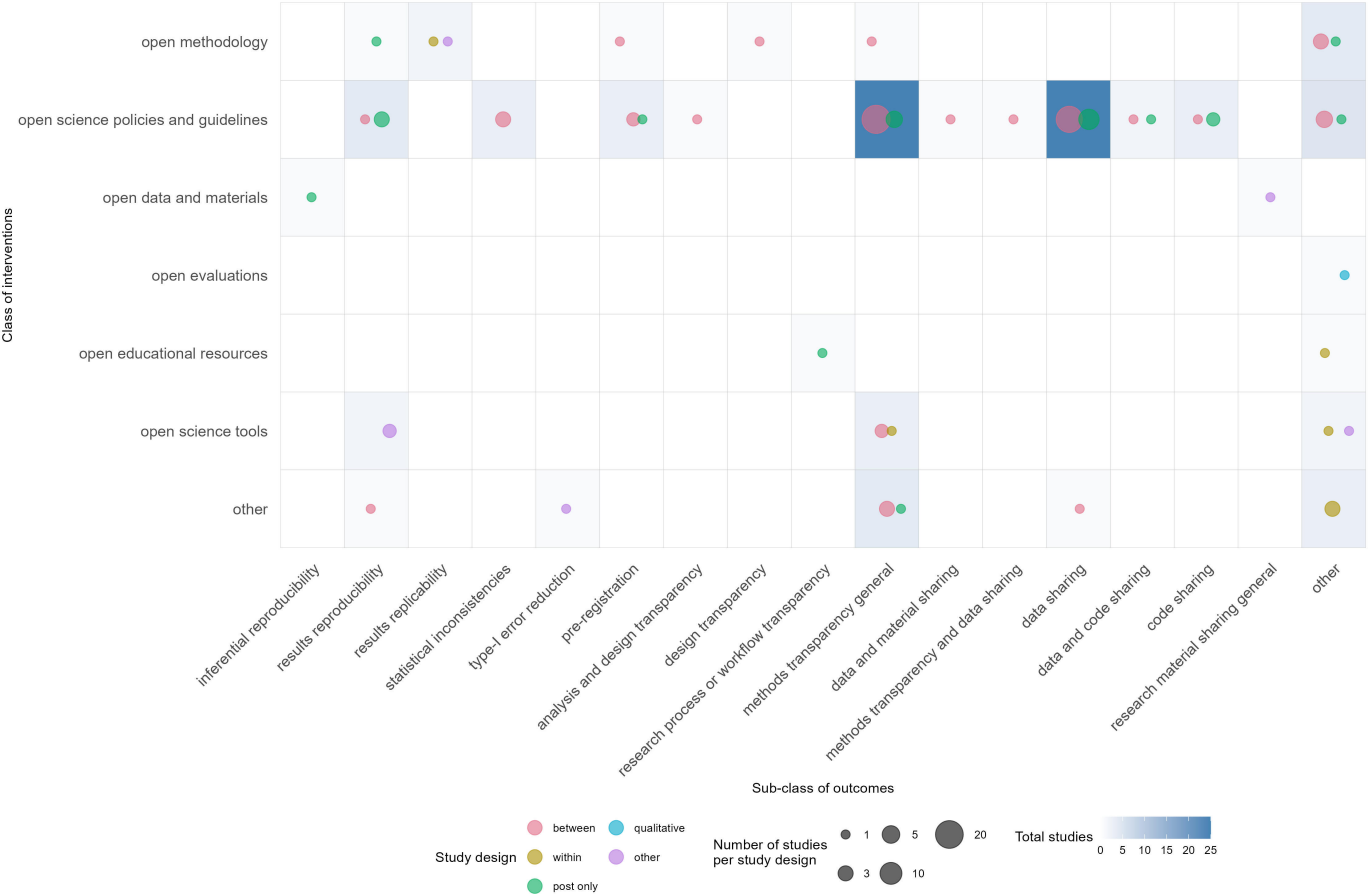
Evidence gap map of interventions and specific outcomes investigated, with information on study designs. Study designs: between = comparative (between-subject comparison); within = comparative (within-subject comparison/repeated measures design); post only = post-intervention (only a post-measurement after the implementation of an intervention and the intervention is explicitly mentioned); qualitative = a qualitative description of what happened during the implementation of an intervention; other = other designs. The size of the bubbles refers to the number of studies that address this particular intervention/outcome combination; the colours refer to different study designs.

**Figure 3.**
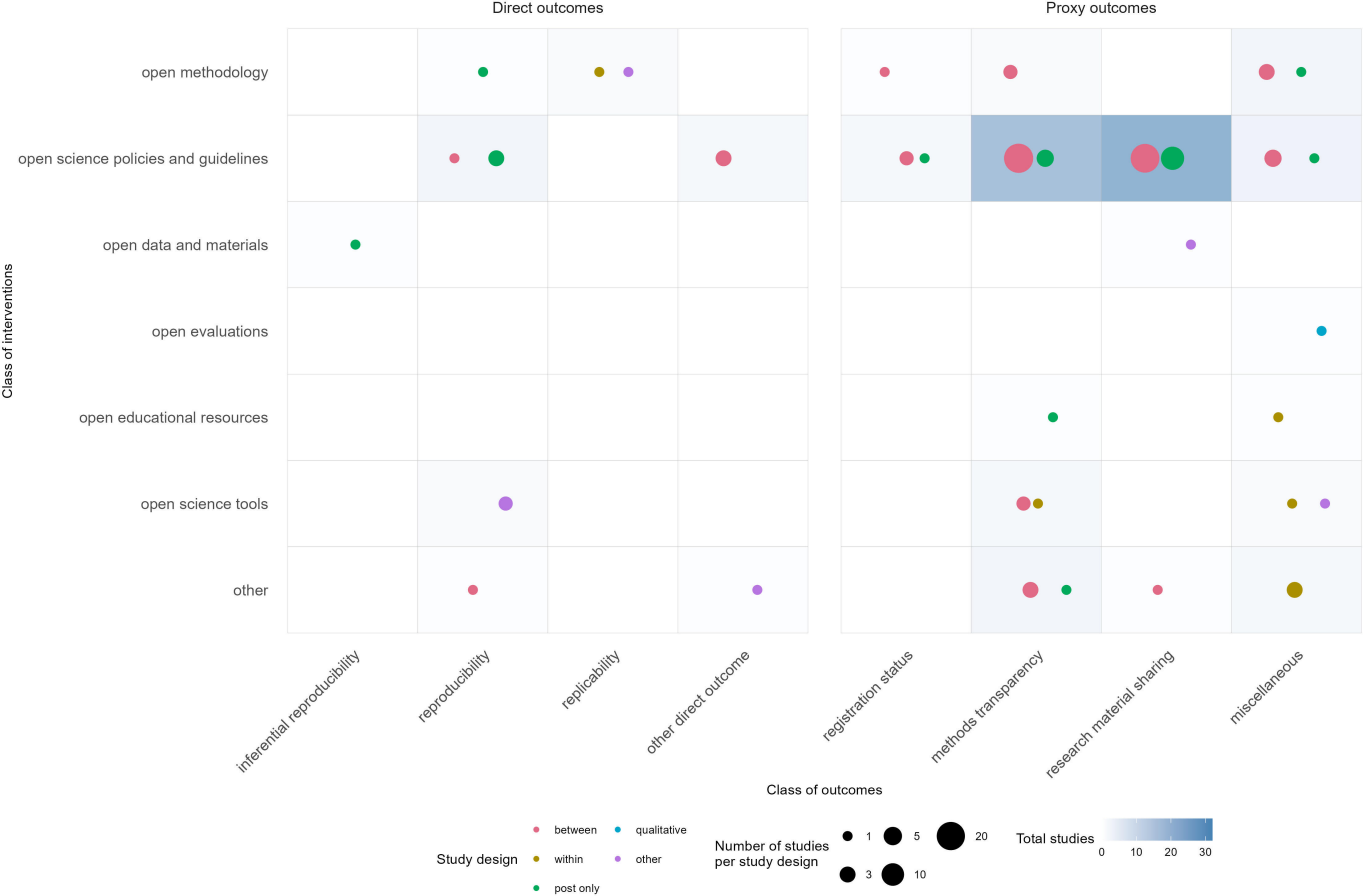
Evidence gap map of interventions and outcome domains investigated, with information on study designs. The left pane shows direct reproducibility outcomes, while the right pane shows proxy outcomes. Study designs: between = comparative (between-subject comparison); within = comparative (within-subject comparison/repeated measures design); post only = post-intervention (only a post-measurement after the implementation of an intervention and the intervention is explicitly mentioned); qualitative = a qualitative description of what happened during the implementation of an intervention; other = other designs. The size of the bubbles refers to the number of studies that address this particular intervention/outcome combination; the colours refer to different study designs.

The evidence gap maps show that most research done according to our scoping review is on the effect of open science policies and guidelines for research transparency—measured as the completeness of reporting; and the effect of open science policies and guidelines on data sharing (figures 2 and 3). A few studies did investigate whether shared data could be used to reproduce research findings. Our evidence gap maps show clear gaps. The most notable gap is the lack of studies investigating the effect of open data and materials (data sharing) on the actual reproducibility and replicability of research. Also, few studies investigated the effect of educational resources and of open evaluations, such as open peer review.

### 3.8. Author-stated direction of effects

**Figure 4.**
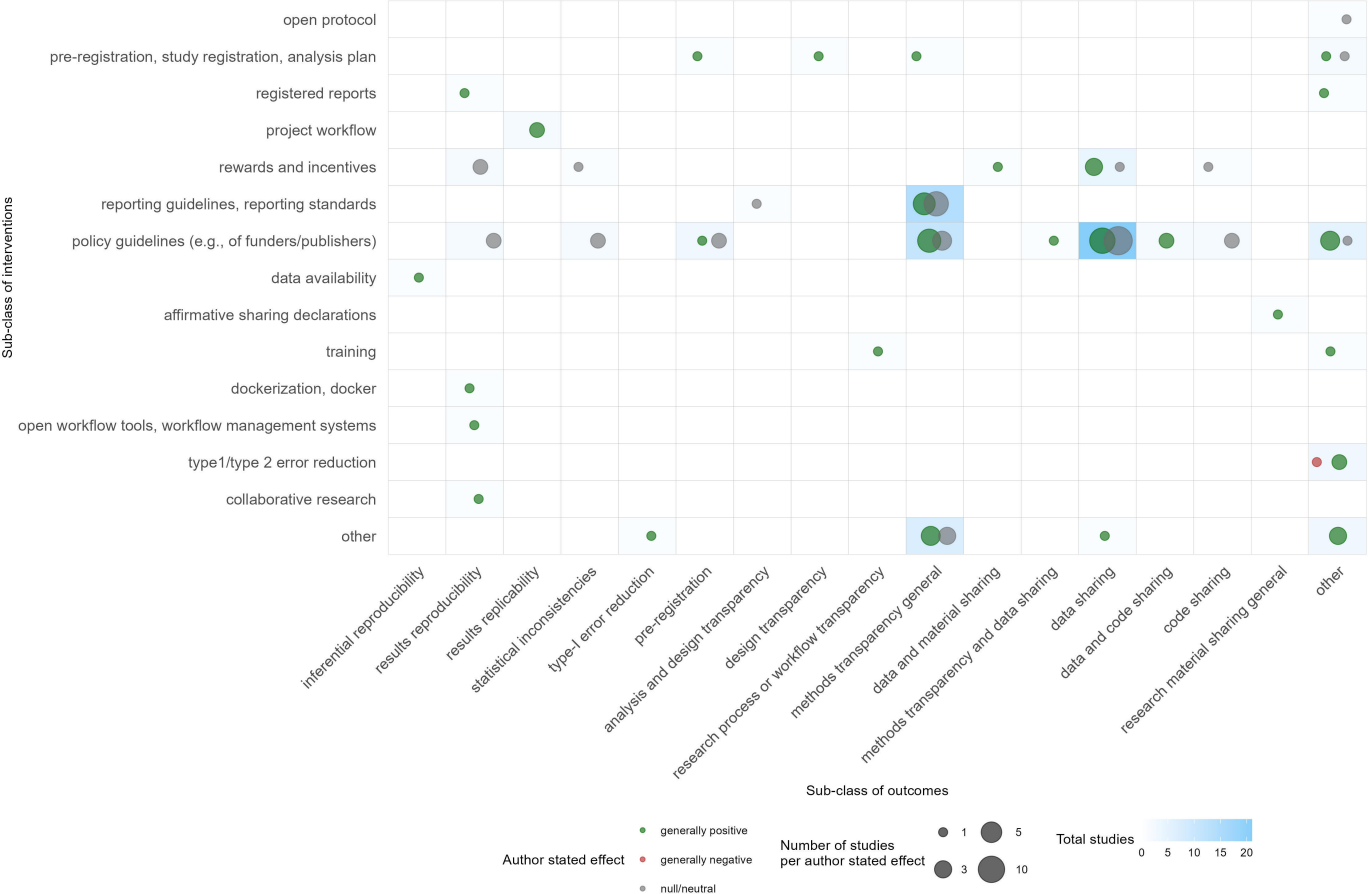
Evidence gap map of interventions and specific outcomes investigated, with information on author-stated effects. Judgment of the effect of the intervention by the authors of the study. Green = generally positive; pink = generally negative; grey = null/neutral. The size of the bubbles was defined by the number of studies that address this particular intervention/outcome combination.

In 60 of 105 research conclusions, the authors of the publications rated the effect of the interventions they investigated positively (figure 4). The authors concluded that these interventions did lead to more reproducibility (or to better methods of transparency, more data/code sharing or more pre-registration as proxies for reproducibility). Forty-three conclusions were neutral, meaning there was no effect detected or authors concluded that the intervention was not effective. Only one study had a negative conclusion, meaning that the intervention had unintended negative consequences, however only regarding a proxy outcome (*p*-value calibration leading to an increased type II error rate) [79].

### 3.9. Disciplinary scope of interventions and temporal distribution

**Figure 5.**
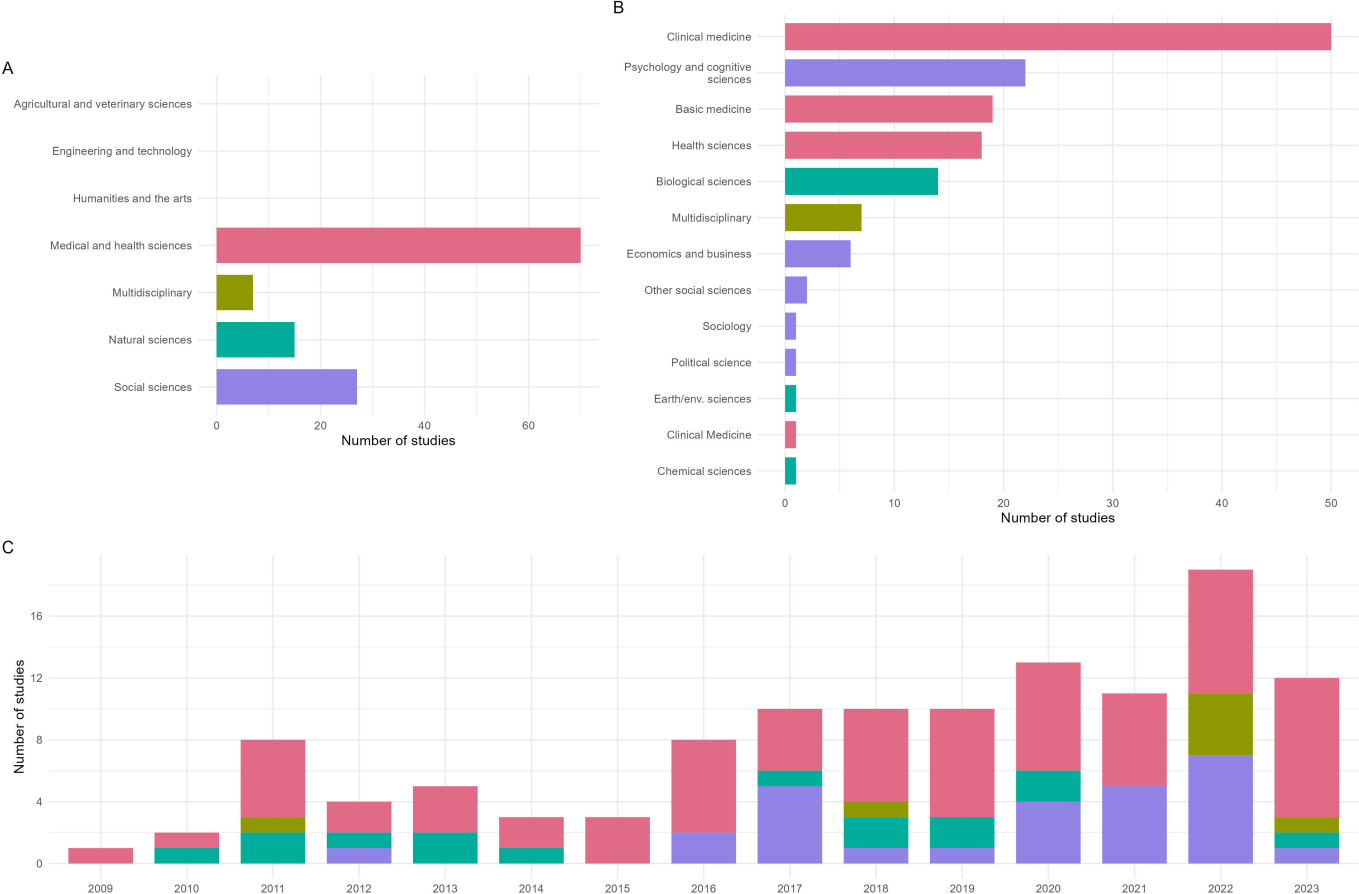
Disciplinary distribution and temporal trends of included intervention studies. Disciplines were coded according to the Frascati manual discipline ’Fields of Science and Technology’ [125]. A. Number of studies according to Frascati Field of Science and Technology Classification schema top-level ‘Disciplines’. B. Number of studies according to Frascati second-level ‘Knowledge Fields’, with respective top-level categories indicated by colour. C. Number of studies over time, with respective top-level categories indicated by colour. Note that each study may cover more than one discipline/field of knowledge. In these cases, the studies are counted fully for each discipline/field of knowledge, and hence overall numbers add up to more than our included number of studies.

Figure 5 presents the disciplinary distribution and temporal trends of our included studies. The majority of studies investigated interventions in the medical and health sciences, with notable contributions from the natural sciences and social sciences. Clinical medicine is by far the most covered area, followed by psychology/cognitive sciences; health sciences; basic medicine; and biological sciences. Multidisciplinary studies (where three or more disciplines are addressed) as well as economics/business are also well represented. The temporal distribution of these studies, with the first published in 2009, and running until 2023 (the time of our database search and retrieval), shows a marked increase in the number of studies over time, peaking in 2022 and 2023 (a partial year given the cut-off represented by the date of our database results retrieval in August 2023). The overall trend indicates a growing number (with a notable recent acceleration) of intervention studies addressing reproducibility and replicability across various disciplines, and an overall predominant focus on clinical medicine, psychology and cognitive sciences, and other health or biological fields.

## 4. Discussion

The aim of this scoping review was to gain an overview of formal investigations of interventions to enhance reproducibility and replicability in research, with an emphasis on those related to open science. Of the 105 distinct studies we included, only 15 directly measured the effect of an intervention on reproducibility or replicability, while the other research questions addressed a proxy outcome, such as data sharing, methods transparency or pre-registration. Thirty research questions were non-comparative and 27 were comparative but used observational cross-sectional designs, precluding any causal inference. Despite studies investigating a range of interventions, employing a range of intervention methods and addressing various outcomes, our findings indicate that in general the evidence base assessing how open science interventions improve reproducibility of research remains remarkably limited in many respects.

### 4.1. State of the evidence on effectiveness of interventions

Despite an ongoing conversation throughout academia regarding the importance of reproducibility and replicability, and the need to intervene to enable more robust and transparent research practices, studies testing interventions in these areas remain relatively scarce. Save for two larger clusters of studies focused on efficacy of data-sharing policies and reporting guidelines, many interventions have been investigated by fewer than five studies and often only by one or two studies. Moreover, these studies have mostly sub-optimal designs for determining clear causal effects; only six studies used a randomized controlled design while most used observational and non-randomized designs. Regarding outcomes, only 15 studies focused on effects related to reproducibility/replicability directly, with others targeting proxy outcomes mostly related to transparency of reporting or sharing of research materials. While the latter are necessary building blocks for enabling reproducibility, they do not directly measure any effects on the robustness of results. Interventions that did focus on reproducibility/replication directly had mixed results, highlighting the need for experimental research specifically designed to test interventions under controlled conditions to provide more definitive evidence on what makes research reproducible.

Interventions that improve transparency, such as data and code sharing, versioning of software, using open software, or using reporting checklists, may be seen as inherently good research practices or as prerequisites for any form of reproducibility. That may mean that it is not necessary to provide solid evidence based on randomized controlled trials. However, as many proposed, interventions in this space may increase workload and costs for researchers, editors and other stakeholders; it is important that we know these resources are being spent on practices that are evidenced as being effective and the magnitude of their effect. We also assessed how the authors of the included articles interpreted their studies. Negative consequences of interventions were generally not reported (e.g. we did not find that something led to less data sharing compared to usual). As this is a scoping review aimed at a general overview of the field and encompasses a wide variety of cross-disciplinary research designs, we did not formally assess the methodological quality of the included research, beyond the comparative limitations of general methodologies for assessing interventions (e.g. randomized versus observational; controlled versus uncontrolled) and therefore are not able to firmly draw conclusions about the quality of evidence for the interventions retrieved.

Lack of research under controlled conditions and a focus on proxy measures rather than reproducibility/replicability themselves may impact the overall relevance of evidence in this area. This limits the extent to which we can currently conclusively discern whether interventions aimed at improving research reproducibility and replicability are actually working. Still, most authors (60 out of 105) concluded positively about the effect of the intervention tested. The positive to neutral nature of the author-stated claims regarding efficacy found in our review may be ironically indicative that meta-research at present may suffer from possible selective reporting and publication bias [126–128] or over-optimistic interpretation of the results [129]. However, the interventions that we retrieved may not be very likely to decrease the level of reproducibility. As such, the positive statements may also be an indication of the causal links between some interventions and reproducibility, such as that using a reporting checklist is likely to lead to (sometimes slightly) better reporting.

### 4.2. Disciplinary coverage and methodological challenges

Alarm about perceptions of a ‘reproducibility crisis’ were initially fuelled by concerns from a few epistemic communities, especially the health and behavioural sciences. Our findings indicate that research into reproducibility has indeed focused on research within these communities. This may suggest that other fields do not face the same degree of challenges to reproducibility, are less engaged with these issues, or find reproducibility to be less relevant for their research (e.g. in the areas of humanities or qualitative research [23,130,131]).

The limited evidence, with gaps in some areas and a complete lack of findings in others, could be attributed to several different causes. Although the debate around these topics is increasing in prominence, research may lag behind these discussions. Considerable time may need to pass before these explicit calls for more research can be met [32], and funding is made available at the scale and scope required for proper investigation. We also believe that opinions on the potential effectiveness of certain practices or tools may often be widely repeated, but poorly evidenced in the actual empirical literature.

### 4.3. Limitations

The primary method for searching articles involved using databases with strict selection criteria. Despite supplementing this with snowballing and searches of secondary literature, it remains quite possible that our review did not include all the available evidence. In addition, although our study team encompassed many disciplines, nonetheless our own understandings of reproducibility undoubtedly shaped the search strategy and data extraction. This may lead to slightly different findings if another team were to replicate our scoping review. However, all experts we contacted did not provide any additional relevant studies that we had not retrieved meaning it is unlikely we overlooked a significant corpus of additional research. Therefore, we believe that our scoping review provides a comprehensive overview of the interventions that have been investigated in this area and of the study designs that have been used to do so.

Related to this, we also have to acknowledge that the search date is more than a year (August 2023) before the peer-reviewed publication of this scoping review (2025). At the time of submission, we checked whether updating the search strategy would be feasible given the limited resources. We estimated that a search update would result in approximately 30% of new items to screen. It can be expected that this will result in some new studies. For example, one of the peer reviewers pointed us to a study that compared the levels of statistical inconsistencies between journals that implemented StatCheck and those that did not [132], and we retrieved through EPPI Reviewer and OpenAlex a preprint concluding that providing article templates does not improve completeness of reporting [133]. However, outdated searches are a recurring problem and we believe that the snapshot we provide still is an accurate reflection of what kind of research is done in this field and a basis for future updates to this review. Furthermore, at least two additional systematic reviews (protocols found here: https://osf.io/7kmxz and https://osf.io/pg3ny) have already benefitted from this scoping review and will further expand the body of evidence.

Discussions about terms and definitions accompanied us throughout the process due to the immense variety in language around reproducibility, open science and related concepts. This variety was captured by the multidisciplinary nature of our team and the broad scope of our review. Additionally, the reporting of some of the included studies allowed for multiple competing interpretations. Barriers like jargon and unclear methodological specification may have limited or obscured our understanding of some findings. Also, not all interventions we describe may be universally regarded as Open Science practices. We all carry our own beliefs and expect to find a scarcity of evidence for most interventions investigated. Hence, despite our best efforts to maintain balance, the combination of our specific perspectives and the width of the included studies have likely shaped our study findings.

Another major limitation is that the screening and data extraction were done by one person for each included study. The lack of clarity of the included studies and the different perspectives of the multidisciplinary team may have led to some variability in extractions. However, we aimed to minimize this through constant communication between team members in weekly meetings and consistency checks across all extractions by the senior author.

### 4.4. Future directions and implications for policy and practice

Investigating the meaning of reproducibility and replicability across contexts may facilitate the understanding of what improves reproducibility/replicability and under which circumstances these improvements can be expected. Therefore, this work should be built upon with a more granular assessment and synthesis of the given studies. Questions for future research include: which specific interventions show the most promise as being effective and therefore merit further investigation? Is there a difference in effect when policies are mandatory versus optional? What characterizes the interventions that are reported to potentially be effective? How does the efficacy of different interventions vary across different stakeholders or implementing institutions? As these questions require an in-depth qualitative and quantitative analysis that would go beyond the aim of this scoping review, we aim to address some of these questions in a future study. Our findings, perhaps above all else, demonstrate a substantial need within the meta-research community for more thorough and rigorous investigations into the effectiveness of interventions aiming to increase levels of reproducibility and replicability. It is striking that researchers, policymakers, journals and other stakeholders are often content to implement large-scale changes without rigorous exploration of their efficacy. More specifically, we call for increased use of standardized and robust experimental designs and the development of a limited set of relevant outcomes that should be investigated in all these studies (core outcome set). Implementing such studies will often involve nuanced approaches involving various stakeholders—including institutions, journals, funders and researchers—who traditionally may not be accustomed to scrutinizing their processes through rigorous experimentation. These groups must become more receptive to subjecting their processes to investigation, as well as sharing data for experimental or observational research [134,135]. Given the previously mentioned observation about possibly over-optimistic reporting or possible selective reporting, we highlight the need for more ‘red-teaming’ [136] of interventions—with researchers less invested in positive outcomes participating.

Given the crucial need to better understand how ‘epistemic diversity’ influences the meaning, relevance and feasibility of reproducibility research [130,137], a corollary of our findings is that further exploring the effectiveness of reproducibility interventions across diverse research contexts is essential. This could be facilitated by global or multi-stakeholder collaborations aimed at developing a common framework for assessing reproducibility and replicability, thereby enhancing the generalizability of findings where possible. Furthermore, expanding the range of disciplines involved in reproducibility research could provide broader insights and innovations, especially regarding which interventions can be implemented at a cross-disciplinary level (using economies of scale) and which require detailed discipline-specific applications.

All this requires funding and resources, of course. Policymakers, funders and institutions, but also publishers and scholarly societies, should consider supporting these lines of inquiry through dedicated funding streams and in-kind access to data and systems for experimentation. Such support would foster more interdisciplinary approaches and help break down the silos that often separate research communities. Towards the latter, further recognizing and supporting 'research on research' as a distinct field with its own conferences, journals and funding lines will also help. Doing so may enable immediate returns on investments by reducing wasted resources dedicated to ineffective interventions throughout the research landscape.

## Data Availability

The data and code are freely accessible through OSF: [138] Supplementary material is available online [139].
